# Clinical pharmacokinetics of capecitabine and its metabolites in colorectal cancer patients

**DOI:** 10.1016/j.jsps.2022.02.019

**Published:** 2022-03-02

**Authors:** Saeed Alqahtani, Rawan Alzaidi, Abdullah Alsultan, Abdulaziz Asiri, Yousif Asiri, Khalid Alsaleh

**Affiliations:** a Department of Clinical Pharmacy, College of Pharmacy, King Saud University, Riyadh, Saudi Arabia; b Clinical Pharmacokinetics and Pharmacodynamics Unit, King Saud University Medical City, Riyadh, Saudi Arabia; c Department of Clinical Pharmacy, College of Pharmacy, King Saud University, Riyadh, Saudi Arabia; d Department of medicine, Oncology center, College of medicine, King Saud University, Riyadh, Saudi Arabia

**Keywords:** Capecitabine, Xeloda, PK, Metabolites, Colon Cancer

## Abstract

**Background:**

Capecitabine is one of the fluoropyrimidine anticancer agents which is extensively used in the management of colorectal cancer. We have noticed a discrepancy between the doses we are using in our patients and the recommended dosing regimen. Thus, this study aims to assess the pharmacokinetic parameters of capecitabine and its metabolites in colorectal cancer patients and report some clinical outcomes.

**Methods:**

This study is a prospective observational pharmacokinetic study. It was conducted at the Oncology Center at King Saud University Medical City. The study included adult patients who received capecitabine for any stage of colorectal cancer. Blood samples were collected following the oral administration of capecitabine. Capecitabine and its metabolites concentration in plasma were determined using HPLC and pharmacokinetic parameters were estimated using PKanalix software.

**Results:**

The study included 30 colorectal cancer patients with a mean age of 58 ± 9.5 years and ECOG Performance Status of 0–1. 60 % of the patients were in stage IV. The average total daily dose was 1265 ± 350 mg/m^2^/day. C_max_ for capecitabine was 5.2 ± 1.3 μg/ mL and T_max_ was 1 ± 0.25 h. AUC_last_ for capecitabine was 28 ± 10 μg.h/ mL. Vd_obs_ and Cl_obs_ for capecitabine were 186 ± 28 L and 775 ± 213 mL/min, respectively. Calculated half-life (t_1/2_) was 2.7 h. Half of our patients showed partial tumor response and 20% showed stable disease. Only two patients had to discontinue the treatment because of the toxicity.

**Conclusion:**

Despite using lower doses, capecitabine and its metabolites parameters were found to be similar to previous studies except for the longer half-life found in our patients. In addition, lower doses of capecitabine showed acceptable response rate which might indicate that higher doses are not always necessary to achieve desired therapeutic effect.

## 1 Introduction

Capecitabine is an oral fluoropyrimidine nucleoside metabolic inhibitor anticancer agent. It is currently approved for adjuvant treatment in patients with Dukes’ C colon cancer; for metastatic colorectal cancer as first-line monotherapy when treatment with fluoropyrimidine therapy alone is preferred; and for metastatic breast cancer as monotherapy for patients resistant to both paclitaxel and an anthracycline-containing regimen or in combination with docetaxel after failure of prior anthracycline containing therapy (Miwa et al., 1998, Reigner et al., 2001). The standard approved dose for these indications is 1250 mg/m^2^ orally twice daily, for 14 consecutive days in 3-week cycles (Miwa et al., 1998, Reigner et al., 2001).

Capecitabine is rapidly absorbed as a prodrug which undergoes activation. The activation process starts with capecitabine hydrolysis by hepatic carboxyl esterase to 5′-deoxy-5-fluorocytidine (5′- 5′-DFCR) (Miwa et al., 1998, Reigner et al., 2001). Then, 5′-DFCR is degraded by cytidine deaminase which is found in tumor cells as well as healthy liver cells to 5′-deoxy-5-fluorouridine (5′-DFUR) (Miwa et al., 1998, Reigner et al., 2001). Subsequently, 5′-DFUR is hydrolyzed by thymidine phosphorylase (TP) to the active molecule 5- Fluorouracil (5-FU) (Schuller et al. 2000). TP is an enzyme that could be found in normal cells as well as tumor cells (Schuller et al. 2000). Fortunately, TP activity in tumor tissue is approximately four times that in adjacent healthy tissue resulting in decreasing 5-FU effect on normal cells (Schuller et al. 2000).

Capecitabine and its metabolite pharmacokinetics were found to be linear and consistent over time when they were studied in a dosage range of 500–3500 mg/m^2^/day in cancer patients (Reigner et al. 2001). However, when the dose was increased, the area under the curves (AUCs) of 5′-DFUR and 5-FU increased higher than the proportion and AUC of 5-FU was 34% higher on the last day in comparison with the first day of the cycle (Reigner et al. 2001).

Colorectal cancer is considered the most common type of cancer in Saudi male patients and the third common type of cancer in female patients with up to 73% of cases diagnosed at late stage (Alyabsi et al. 2020). Currently, capecitabine is considered one of the most important cancer treatments especially for metastatic colorectal cancer patients. Being an oral medication, it provides a convenient alternative for the patients with comparable efficacy and apparently more tolerability than IV 5- FU (Van Cutsem et al., 2000, Hoff et al., 2001). Additionally, it showed lower cost than 5-FU in several pharmacoeconomic studies (Twelves et al., 2001, Cassidy et al., 2006).

Although the recommended dose of capecitabine is 1250 mg/m^2^ orally twice daily, we noticed that our patients are receiving less than this dose mainly because of the side effects. The use of variable total dose of capecitabine results in substantial interpatient variability in drug exposure. Therefore, because of the limited data of capecitabine pharmacokinetic in Saudi population, we aimed in this study to assess clinical pharmacokinetics and disposition of capecitabine and its metabolites 5′-DFUR, 5′-5′-DFCR and 5-FU in colorectal cancer patients and to determine the initial and steady state PK parameters including AUC, Cmax, Vd, and CL. In addition, we report some clinical outcomes.

## 2 Methods

### 2.1 Study design and subjects

This was a prospective pharmacokinetic study. This study was conducted at the Oncology Center at King Saud University Medical City/King Khalid University Hospital. It included patients who were admitted to the Oncology Center and received capecitabine for any stage of colorectal cancer. Patients received oral capecitabine, divided into two daily doses for 14 consecutive days as anticancer monotherapy at each cycle. Cycles were repeated every three weeks (14 days treatment, seven days break) for a total of six cycles. Inclusion criteria were as follows: all adult patients, male and female, ≥ 18 years old; white blood cell count greater than 4 x10^9^/l; no renal impairment as judged by standard biochemical parameters (plasma creatinine < 120 μmol/l); and no hepatic impairment (bilirubin < 5 μmol/l and alaninaminotransferase < 30 IU/l). The study complied with legal requirements and the Declaration of Helsinki, and was approved by the King Saud University Medical City IRB. Each patient has provided informed consent to participate in the study.

### 2.2 Blood sampling

Blood samples of 5 mL were taken at 1, 2, 4, 6, 8 and 12 h after drug administration at day 5 of the first cycle, using sodium-heparinized vacutubes. Blood samples were centrifuged, and the plasma was stored in plastic tubes at − 80 °C until analysis.

### 2.3 Analytical method

Extraction of capecitabine, 5′-5′-DFCR, 5′-DFUR, 5-FU from plasma samples was performed by precipitation with acetonitrile. The compounds in the supernatant were separated into two fractions using an automatic solid phase extraction system (Oasis HLB 1 cm^3^, 30 mg packing volume; Waters, Milford, MA, USA): capecitabine, 5′-5′-DFCR, and 5′-DHUR were eluted with methanol (fraction A); and 5-FU was eluted with ammonium acetate (fraction B) (Reigner et al. 1998).

Determination of capecitabine and its metabolites (5′-5′-DFCR, 5′-DFUR, and 5-FU) conducted by using previously published high-performance liquid chromatography (HPLC) method with an external standard method (Ciccolini et al., 2004, Farkouh et al., 2010). The system consisted of a SIL-20AHT autosampler, Shimadzu UV SPD-20A, and an LC-20AB pump connected to a Dgu-20A3 degasser. Data acquisition was achieved by using LC Solution software version 1.22 SP1. Separation of capecitabine, 5′-5′-DFCR, and 5′-DFUR were performed on a Phenomenex Luna C18 column (250 × 4.6 mm i.d., 5 μm; Phenomenex, CA). The mobile phase consisted of water and methanol of HPLC grade purity (50 + 50%, v/v) mixed with 0.005 M disodium-hydrogen phosphate, adjusted to pH 8.0 with phosphoric acid; flow rate at 0.5 mL/min. The detection of capecitabine was at 305 nm, and for 5′- 5′-DFCR, and 5′-DFUR, the detection was at 240 nm.

For 5-FU from the fraction B, the separation was performed on a Phenomenex Luna C18 column (250 × 4.6 mm i.d., 5 μm; Phenomenex, CA). The mobile phase consisted of a potassium phosphate salt solution (0.05 m) + 0.1% triethylamine (TEA); flow rate 0.4 mL/min. Detection of 5-FU and 5-bromouracil were performed at 254 nm.

Standard curves for capecitabine, 5′-5′-DFCR, 5′-DFUR, and 5-FU were prepared using plasma samples from healthy volunteers and spiked with those agents in the range 25–10,000  ng/mL. Extraction yield was calculated by comparing peak heights in five extracted samples (from lower and higher range) comparing to standard solutions. Extraction yield was 93%. The method was valid with a lower limit of quantification of 50 ng/ mL for all tested compounds, and an inter-day coefficient of variation of 5.5% when analyzed in spiked plasma samples.

### 2.4 Pharmacokinetic analysis

The pharmacokinetic parameters of capecitabine and its metabolites (5′-5′-DFCR. 5′- DFUR, and 5-FU) were determined from the concentration–time data using non-compartmental model of PKanalix software. The following parameters were estimated. Maximum plasma concentrations (C_max_) and time of their occurrence (T_max_) were determined from the observed highest concentration and its occurrence, respectively. AUC_0-t_ is the area under the curve from time 0 to the last sampling time (t last) at which the concentration could be measured (C_last_). MRT_last_: mean residence time is from 0 to t_last_ (h). Volume of distribution (Vd_obs_) and clearance (Cl_obs_) were calculated for capecitabine but not for the other metabolites.

## 3 Results:

### 3.1 Study subjects

The study included thirty patients (fourteen females and sixteen males), their mean age was 58 ± 9.5 years, ECOG Performance Status 0–1, mean body weight was 72.2 ± 16.2 kg, mean height was 164.4 ± 13.2 cm and mean body surface area was 1.78 ± 0.4 m^2^. All patients had adequate hematological, renal and hepatic functions. The patients’ characteristics are summarized in Table 1. Of these patients, 60 % of the patients were in stage IV of the colorectal cancer, 23.3% were in the stage III, and 16.6% were in stage I and II. The average total daily dose was 1265 ± 350 mg/m^2^/day. 60% of the patients were on 1000 mg twice daily. Oxaliplatin, bevacizumab, and irinotecan were the most common combinations given with capecitabine.

**Table 1:** Baseline demographic and disease characteristics of patients included in the analysis.

Characteristics	Mean (S.D)
Age, years	58 (9.5)
Gender, % male/%female	53/47
ECOG	0–1
Weight, kg	72.2 (16.2)
Height, cm	164.4 (13.2)
BMI	27.2 (5.7)
BSA, m^2^	1.78 (0.4)
Total daily dose, mg/m^2^	1265 (3 5 0)
Serum creatinine, μmol/l	61 (25.9)
CrCl, mL/min	102 (33.5)
Albumin concentration, g/l	32.9 (7.6)
AST, IU/l	24 (10.2)
ALT, IU/l	22.6 (21.5)
Direct Bilirubin, μmol/l	2.89 (1.3)
WBC, x10^9^/l	5.6 (2.5)
Hb, g/l	122.5 (18.8)
Platelet, x10^9^/l	226 (1 0 0)
Disease stage, n (%)	
Stage I and II	5 (16.6%)
Stage III	7 (23.3%)
Stage IV	18 (60%)
Other drugs	
Oxaliplatin	9 (30%)
Bevacizumab	7 (23%)
Irinotecan	3 (10%)

### 3.2 Pharmacokinetics analysis

**Fig. 1:**
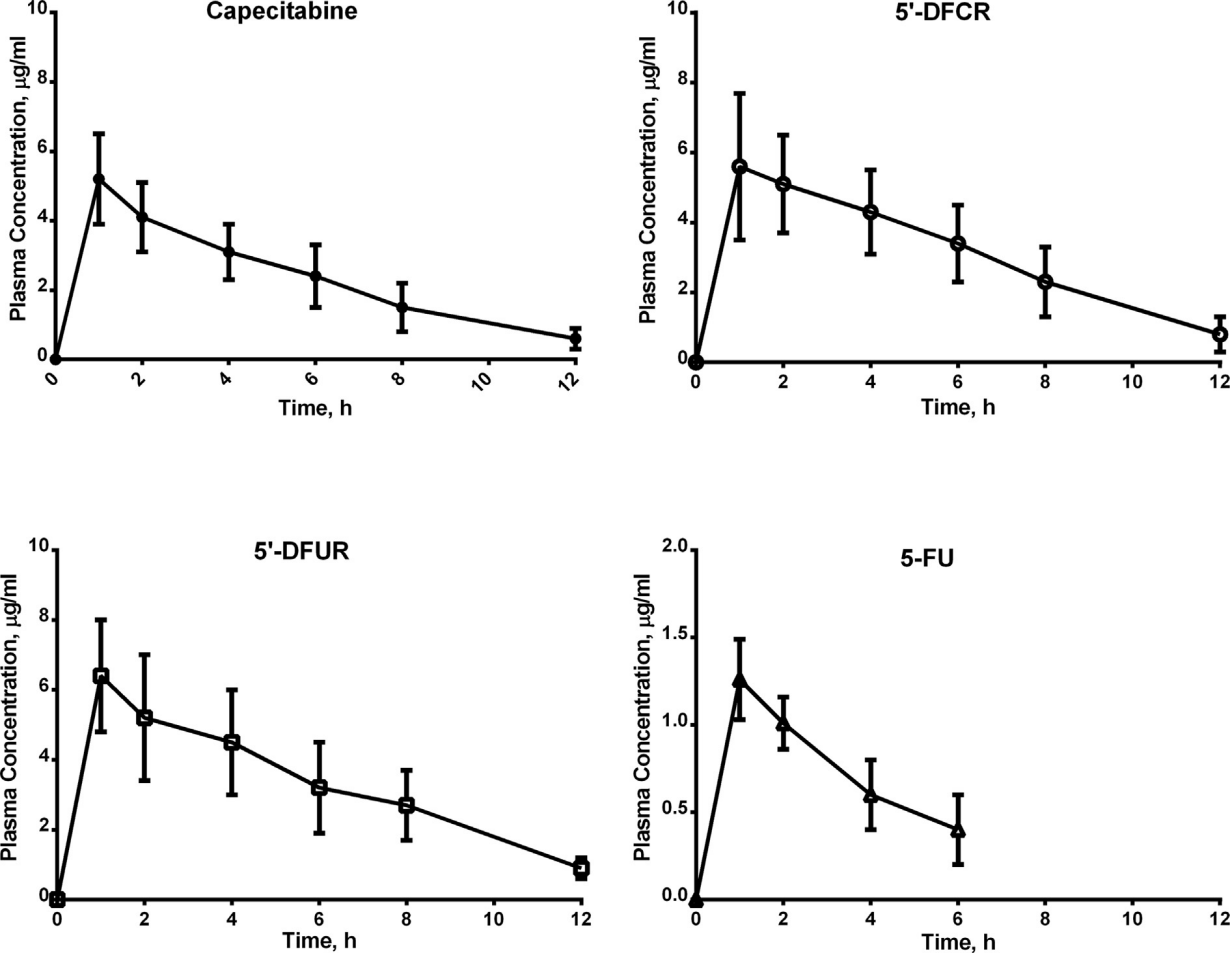
Mean plasma concentration time curves of capecitabine and its metabolites. 5′-DFCR: 5′deoxy-5-fluorocytidine; 5′-DFUR: 5′-deoxy-5-fluorouridine; 5-FU: 5-Fluorouracil.

Table 2 presents the mean ± SD key pharmacokinetic parameters for capecitabine and its metabolites. C_max_ for capecitabine was 5.2 ± 1.3 μg/mL and T_max_ was 1 ± 0.25 h. AUC_last_ for capecitabine was 28 ± 10 μg.h/mL. Vd_obs_ and Cl_obs_ for capecitabine were 186 ± 28 L and 775 ± 213 mL/min, respectively. Calculated half-life (t_1/2_) was 2.7 h. MRT_last_ for capecitabine was 1 ± 0.15 h. The pharmacokinetic parameters for the metabolites are presented in Table 2. Plasma concentration time curves of capecitabine and its metabolites are presented in Fig. 1.

**Table 2:** Pharmacokinetic parameters for capecitabine and its metabolites (Mean ± SD).

**Parameter**	**Mean (S.D)**
**Capecitabine**	
**T_max_ (h)**	1 (0.25)
**C_max_ (μg/mL)**	5.2 (1.3)
**C_last_ (μg/mL)**	0.6 (0.23)
**AUC_last_ (μg.h/mL)**	28 (10)
**Vd_obs_ (l)**	186 (28)
**Cl_obs_ (mL/min)**	775 (2 1 3)
**MRT_last_ (h)**	1 (0.15)
**t_1/2_ (h)**	2.7

**5′-DFCR**	
**T_max_ (h)**	1 (0.3)
**C_max_ (μg/mL)**	5.6 (2.1)
**AUC_last_ (μg.h/mL)**	37.15 (12)

**5′-DFUR**	
**T_max_ (h)**	1 (0.25)
**C_max_ (μg/mL)**	6.4 (1.6)
**AUC_last_ (μg.h/mL)**	39.5 (13)

**5-FU**	
**T_max_ (h)**	1 (0.35)
**C_max_ (μg/mL)**	1.26 (0.23)
**AUC_last_ (μg.h/mL)**	4.4 (2)

### 3.3 Pharmacology data

The aim of this PK study was to determine the pharmacokinetic parameters of capecitabine and its main metabolites in patients receiving capecitabine and other chemotherapy. While we did not set out to evaluate antitumor response, efficacy data were collected in this group of patients and followed-up during the period of treatment. 15 of the 30 patients showed a partial tumor response (which defined as 30% reduction in the total tumor measurement) while six patients showed stable disease. On the other hand, seven patients showed progression in their disease and two patients were unable to complete the treatment course because of the toxicity.

## 4 Discussion

This was the first study that assessed capecitabine clinical pharmacokinetics in Saudi papulation. The study included 30 colorectal cancer patients with good performance status. The majority of patients had an advanced stage disease. The average daily dose of capecitabine was 1265 mg/m^2^ which is approximately half the standard U.S FDA approved dose of 2500 mg/m^2^ daily.

In this study, the C_max_ for capecitabine was 5.2 ± 1.3 μg/mL which was slightly lower than its metabolites 5′-DFCR 5.6 ± 2.1 μg/mL and 5′-DFUR 6.4 ± 1.6 μg/mL. On the other hand, 5-FU had the lowest C_max_ level of approximately 1.26 ± 0.23 μg/mL. Moreover, it was found that, AUC_last_ was larger for 5′-DFCR 37.15 ± 12 μg.h/mL and 5′-DFUR 39.5 ± 13 μg.h/mL than capecitabine 28 ± 10 μg.h/mL. However, 5-FU showed the smallest AUC_last_ of 4.4 ± 2 μg.h/mL.

Additionally, we found that it required 0.75 to 1.25 h for capecitabine and 5′-DFUR to reach the maximum plasma concentration. However, it took its metabolites a slightly longer time ranging from 0.7 to 1.3 h for 5′-DFCR and 0.65 to 1.35 for 5-FU. Similarly, previous studies have shown capecitabine reaching peak blood levels in about 0.75 to 2 h following oral administration of 1000 to 1250 mg/m2 (Miwa et al., 1998, Reigner et al., 1998, Cassidy et al., 1999, Reigner et al., 1999, Twelves et al., 1999, Farkouh et al., 2010, Farkouh et al., 2014).

In this study, capecitabine showed a large volume of distribution with a value of 186 ± 28 L which was unvarying from previous studies, which is indicative for good tissue distribution and low protein binding (Farkouh et al. 2014).

Interestingly, regardless of the lower doses that our patients received (1000 mg total dose), their C_max_ results are consistent with data from previous studies that included around 62 patients showing that after single doses ranging from 1250 to 1255 mg/m^2^, capecitabine mean C_max_ was 2.7 to 4.0 μg/mL (Reigner et al., 1998, Cassidy et al., 1999, Reigner et al., 1999, Twelves et al., 1999). At the same time, it showed lower maximum concentration for 5-FU of 0.22 to 0.31 μg/mL (Reigner et al., 1998, Cassidy et al., 1999, Reigner et al., 1999, Twelves et al., 1999).

Furthermore, TP is the enzyme responsible for converting capecitabine into its active metabolite 5-FU. It was found previously that its activity in tumor cells was 4 times more than normal cells (Schuller et al. 2000) which could explain the low systemic level of 5-FU observed in our study as well as previous studies (Reigner et al., 1998, Cassidy et al., 1999, Reigner et al., 1999, Twelves et al., 1999). Moreover, these low levels might contribute to the lower fluorouracil toxicity risk with capecitabine.

Nevertheless, our patients had generally a good renal function with a mean CrCl of 102 mL/min, they showed lower Cl_obs_ 775 ± 213 mL/min than previous studies 5858 mL/min (Farkouh, Scheithauer et al. 2014).

In addition, t_1/2_ in this study was 2.7 h which is approximately three times earlier reported t1_/2_ ranging from 0.55 to 0.89 h (Reigner et al., 1998, Cassidy et al., 1999, Reigner et al., 1999, Twelves et al., 1999). This might explain how our patients achieved sufficient C_max_ levels with lower doses.

The inter-individual variability in the PK of capecitabine has been noticed in previous studies. It was found to be high around 27 to 89% and is expected to be mostly due to variability in the activity of the enzymes implicated in capecitabine metabolism (Miwa et al. 1998). The inter-individual variability in the C_max_ and AUC of 5-FU was greater than 85% (Miwa et al. 1998). Based on their observations, the standard dosing of capecitabine is so far debatable for geographical differences. Haller et al. published a retrospective analysis of colorectal cancer patients who were treated with capecitabine worldwide, the authors found that patients in the United States “had higher rates of grade 3 and 4 adverse events (relative risk [RR], 1.77)”, which resulted in dose reduction “(RR, 1.72)”, with a high rate of treatment withdrawal “(RR, 1.83)”(Haller et al., 2008, Hirsch and Zafar, 2011). In the TREE trials, TREE-1 started with 150 patients who were treated with 1000 mg/m^2^ capecitabine and oxaliplatin. However, in the subsequent trial - TREE-2, they had 223 patients with 850-mg/m2 capecitabine. The dose was reduced after the data monitoring committee assessed the safety and toxicity of the original study (Hochster et al., 2008, Hirsch and Zafar, 2011). In addition, there is a study labeled as SO14693 of a total of thirty-three patients with solid metastatic tumors, mostly colorectal cancer, that were enrolled consecutively in cohorts of 3 to 6 patients to overall 7, while escalating the doses of capecitabine at 110, 225, 502, 1003, 1331, 1657, and 2083 mg/m2/day orally twice daily for six weeks. When they reached the 1657 mg/m2/day dose, 8 of 12 patients (2 cohorts of 6 patients) experienced grade 3 toxicity; consequently, this dose was considered the maximum tolerated dose. The recommended dose for further trials was 1331 mg/m^2^/day (EMA 2015).

Variances between Caucasian and Japanese patients can be explained as the differences in the therapeutic effect of medications and their adverse events triggered by their genetic difference. However, this is quite questionable when it comes to the differences between the United States and European populations who have comparable genetic profiles (Midgley and Kerr, 2009, Hirsch and Zafar, 2011). Other factors could be the reason for such differences such as body weight, age, sex, and diet, but they still have not been verified.

Moreover, in spite of receiving lower doses, half of our patients showed partial tumor response and 20% showed stable disease. On the other hand, only two patients had to discontinue the treatment because of the toxicity, although they received the average dose and showed average PK parameters comparing to other patients. This might indicate that lowering the dose could still provide a good clinical response with lower toxicity.

## 5 Conclusion

Over the past ten years, capecitabine has been studied as a treatment of colorectal carcinoma, and in general, is considered to have an advantage in terms of overall survival in patients with metastatic colorectal cancer. For the purposes of this investigation, the pharmacokinetic parameters of capecitabine and its metabolites in colorectal cancer patients were reported. Despite using lower doses, capecitabine and its metabolites parameters were found to be similar to other studies except for the longer half-life found in our patients. In addition, lower doses of capecitabine showed acceptable response rate which might indicate that higher doses are not always necessary to achieve desired therapeutic effect. However further researches are needed to conclude this theory.

## Declaration of Competing Interest

The authors declare that they have no known competing financial interests or personal relationships that could have appeared to influence the work reported in this paper.
